# An assessment on epitope prediction methods for protozoa genomes

**DOI:** 10.1186/1471-2105-13-309

**Published:** 2012-11-21

**Authors:** Daniela M Resende, Antônio M Rezende, Nesley JD Oliveira, Izabella CA Batista, Rodrigo Corrêa-Oliveira, Alexandre B Reis, Jeronimo C Ruiz

**Affiliations:** 1 Programa de Pós-graduação em Ciências Farmacêuticas (CiPharma), Laboratório de Pesquisas Clínicas, Escola de Farmácia, Universidade Federal de Ouro Preto, Campus Morro do Cruzeiro, Ouro Preto, MG, 35400-000, Brazil; 2 Laboratório de Imunologia Celular e Molecular, Instituto René Rachou, Av. Augusto de Lima, 1715, Barro Preto, Belo Horizonte, MG, 30190-002, Brazil; 3 Laboratório de Imunopatologia, Núcleo de Pesquisas em Ciências Biológicas, Universidade Federal de Ouro Preto, Campus Morro do Cruzeiro, ICEB II, Ouro Preto, MG, 35400-000, Brazil; 4 Laboratório de Parasitologia Celular e Molecular, Instituto René Rachou, Av. Augusto de Lima, 1715, Barro Preto, Belo Horizonte, MG 30190-002, Brazil; 5 Departamento de Bioquímica e Imunologia, Instituto de Ciências Biológicas, Universidade Federal de Minas Gerais, Av. Antônio Carlos, Pampulha, 6627, 31270-901, Belo Horizonte, MG, Brazil; 6 Pontifícia Universidade Católica, R. Rio Comprido, 4580, Monte Castelo, Contagem, MG, 32285-040, Brazil; 7 Centro Universitário UNA, Instituto de Ciências Biológicas e da Saúde (ICBS), R. Guajajaras, 175, Centro, Belo Horizonte, MG 30180-100, Brazil

## Abstract

**Background:**

Epitope prediction using computational methods represents one of the most promising approaches to vaccine development. Reduction of time, cost, and the availability of completely sequenced genomes are key points and highly motivating regarding the use of reverse vaccinology. Parasites of genus *Leishmania* are widely spread and they are the etiologic agents of leishmaniasis. Currently, there is no efficient vaccine against this pathogen and the drug treatment is highly toxic. The lack of sufficiently large datasets of experimentally validated parasites epitopes represents a serious limitation, especially for trypanomatids genomes. In this work we highlight the predictive performances of several algorithms that were evaluated through the development of a MySQL database built with the purpose of: a) evaluating individual algorithms prediction performances and their combination for CD8+ T cell epitopes, B-cell epitopes and subcellular localization by means of AUC (Area Under Curve) performance and a threshold dependent method that employs a confusion matrix; b) integrating data from experimentally validated and *in silico* predicted epitopes; and c) integrating the subcellular localization predictions and experimental data. NetCTL, NetMHC, BepiPred, BCPred12, and AAP12 algorithms were used for *in silico* epitope prediction and WoLF PSORT, Sigcleave and TargetP for *in silico* subcellular localization prediction against trypanosomatid genomes.

**Results:**

A database-driven epitope prediction method was developed with built-in functions that were capable of: a) removing experimental data redundancy; b) parsing algorithms predictions and storage experimental validated and predict data; and c) evaluating algorithm performances. Results show that a better performance is achieved when the combined prediction is considered. This is particularly true for B cell epitope predictors, where the combined prediction of AAP12 and BCPred12 reached an AUC value of 0.77. For T CD8+ epitope predictors, the combined prediction of NetCTL and NetMHC reached an AUC value of 0.64. Finally, regarding the subcellular localization prediction, the best performance is achieved when the combined prediction of Sigcleave, TargetP and WoLF PSORT is used.

**Conclusions:**

Our study indicates that the combination of B cells epitope predictors is the best tool for predicting epitopes on protozoan parasites proteins. Regarding subcellular localization, the best result was obtained when the three algorithms predictions were combined. The developed pipeline is available upon request to authors.

## Background

Reverse vaccinology uses the genome sequences of viral, bacterial or parasitic pathogens of interest rather than the cells as starting material for the identification of novel antigens, whose activity should be subsequently confirmed by experimental biology approaches [1]. In general, the aim of this approach is the identification of genes potentially encoding pathogenicity factors and secreted or membrane-associated proteins. In this context, specific algorithms suitable for the *in silico* identification of novel surface-exposed and, thus, antibody accessible proteins mediating a protective immune response are used [2].

Pizza and co-workers in collaboration with The Institute for Genomic Research (TIGR) provided the first example of a successful application of the reverse vaccinology approach [3]. They described that *in silico* identification of vaccine candidates against *Neisseria meningitides* serogroup B, which is the major cause of sepsis and meningitis in children and young adults, could be effective, while conventional approaches to obtain a vaccine had failed for decades.

New powerful genomic technologies have increased the number of diseases that can be addressed by vaccination, and have reduced the time of discovery research and vaccine development [1]. Nowadays, it costs US$ 200–400 million to research, develop, manufacture and launch a new vaccine on the global market [4]. With the use of reverse vaccinology, time and cost spent on the search of new vaccine targets are significantly reduced.

Immunoinformatics is an emerging application of bioinformatics techniques that focuses on the structure, function, and interactions of the molecules involved in immunity. One of its main goals is the *in silico* prediction of immunogenicity at epitope level. Recently developed *in silico* tools and databases can be used to identify, characterize or predict antigen epitopes recognized by T- and B-lymphocytes, cells that play significant roles in infection and protective immunity [5].

Epitopes are the minimal essential units of information derived from self and nonself proteins that stimulate cellular (T-cell) and humoral (B-cell) immune responses. T-cells recognize T-cell epitopes that are derived from endogenous and exogenous proteins and presented in the cleft of MHC class I or MHC class II molecules at the surface of antigen presenting cells to the T-cell receptor. After the activation of CD8+ T cells or CD4+ T cells, respectively, cellular events, such as citotoxicity and cytokine secretion, will occur. B-cells also recognize epitopes, but generally intact proteins. B-cell epitopes can be linear, contiguous amino acids, or discontinuous amino acids that are brought together in folded proteins. After activation, B-cells differentiate into plasmocytes and start secreting antibodies. B- and T-cell responses are called humoral and cellular adaptive immune responses, respectively, and they inform the immune system that a bacteria, virus, or parasite is present [6].

The subcellular localization of the protein is also important to investigate, as immunogenic proteins have to be in contact with T- and B-cells in order to elicit a protective immune response. In other words, correct subcellular localization is of great significance to the functional analysis of proteins [7]. Therefore, various prediction methods have been developed to predict proteins’ subcellular location in the recent decades [8]. Prediction methods to identify the subcellular location of proteins can be classified into two categories: one is based on the recognition of protein N-terminal sorting signals [9] and the other is based on amino acid composition [10]. The predictors then combine these features with machine-learning techniques to decide which is the most probable location [11,12].

A large variety of machine-learning techniques are commonly used in bioinformatics, including artificial neural networks (ANNs) [13], hidden Markov models (HMMs) [14] and support vector machines (SVMs) [15]. ANNs and SVMs are ideally suited to recognize non-linear patterns, which are believed to contribute to, for instance, peptide-HLA-I interactions [16]. In an ANN, information is trained and distributed into a computer network with an input layer, hidden layers and an output layer all connected in a given structure through weighted connections [13]. Finally, HMMs are well suited to characterized biological motifs with an inherent structural composition, and have been used in the field of immunology to predict peptide binding to major histocompatibility complex (MHC) class I molecules [17].

The use of database system has been constant in the life of researchers and professionals in several fields. Conceptually, a database should be able to provide an easy access to experimental results and lexical surveys, preventing redundancy and wasteful duplication of research data. A well-designed database should also be able to provide support to researchers, facilitating guided searches for novel correlations in data. On the other hand, a poorly designed database makes the data mining process difficult and the new data integration infeasible for regular users [18] and in this perspective, the rebuilding and redesigning processes are frequent [18,19].

The current challenge of modern biology is to unravel and understand the complex system of biological organization and to signal in all of its details at a molecular level. An essential part of this process goes through bioinformatics, particularly the use of management systems, and relational databases applied to biological data. Biological data reside in specialized databases that represent different data interpretation stages or different facets of biological phenomena [20]. Also, biological data present a particularity: they are highly complex when compared with data from most of other applications. Thus, definitions of such biological data must be able to represent a complex substructure of data as well as their relationships, and also ensure that no information is lost during the biological data modeling. The data model must be able to represent any level of complexity in any data schema, relationship, or schema substructure and not just in a hierarchical, binary, or tabular data format [21].

**Figure 1:**
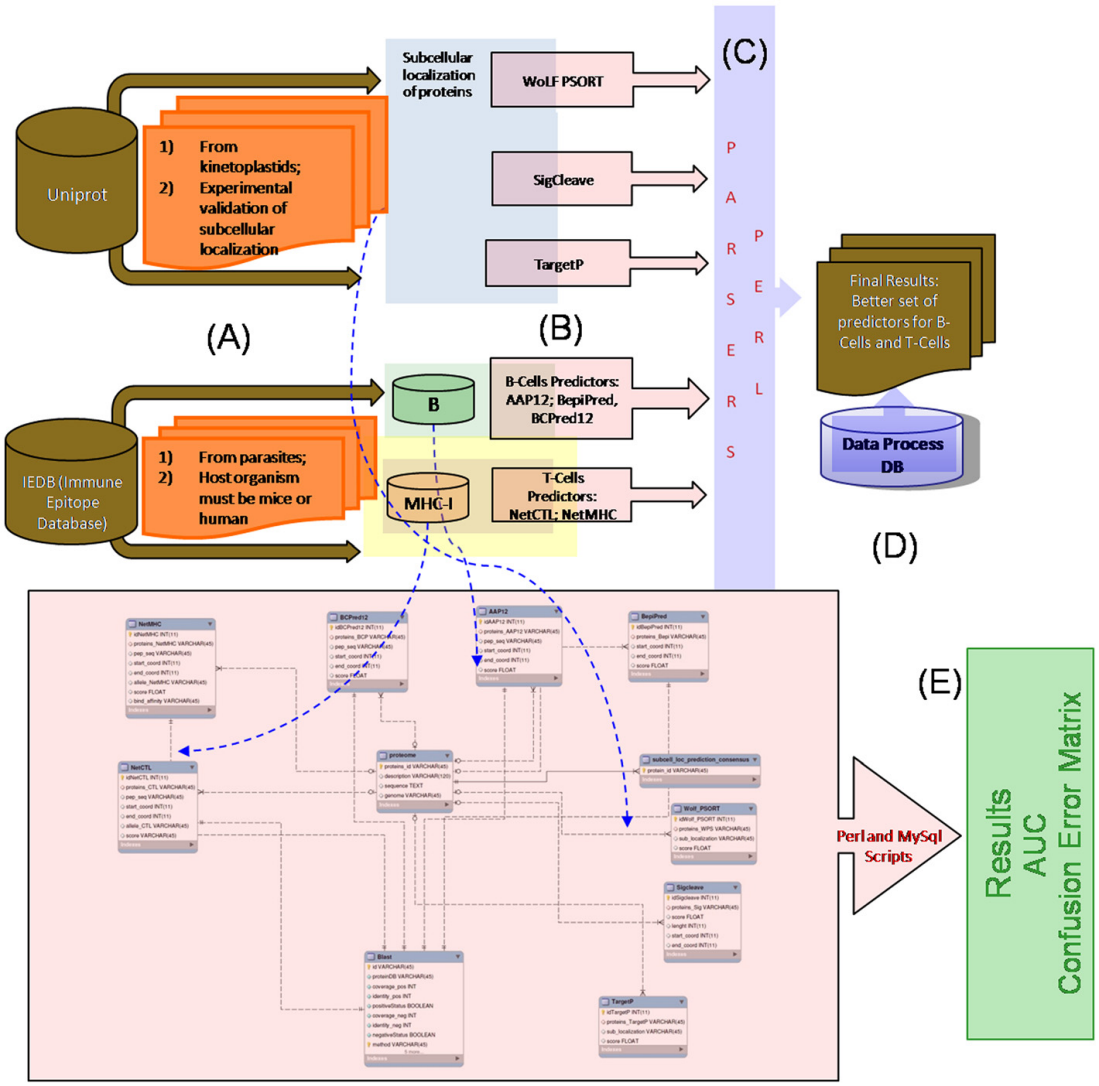
**The methodology flowchart used to develop this work. A** - Construction of the experimentally tested epitopes database, obtained on the curated Immune Epitope Database and Analysis Resource (IEDB) and on Uniprot. **B** - Algorithms used to predict B-cell epitopes, MHC-I epitopes, MHC-II epitopes and subcellular location of proteins. **C** - Parsers developed to extract the results. **D** - Construction of the relational database to integrate the results. **E** - Analysis of the results in the framework of AUC and confusion error matrix. PERL, Practical and Extraction Report Language; SQL, Structured Query Language.

The main objective of this present work was to build a database-driven epitope prediction method capable of accurately predicting parasite B- and T-cell epitopes, as well as subcellular localization of parasites proteins (Figure 1). The interface language used was standard SQL (Standard Query Language) and several built-in functions were implemented, but are not limited to, the following: a) parse algorithms predictions and storage of experimental validated and predicted data; and b) evaluation of algorithm performances.

## Results

### MHC-I epitopes prediction

Several approaches that predict peptide binding to MHC molecules have been published [22]. In this study we opt for two currently available algorithms, NetCTL [22] and NetMHC [23]. Our choice for these MHC class I epitope prediction algorithms was made in terms of ISI citation indexes and regarding their availability for download and local machine implementation.

**Figure 2:**
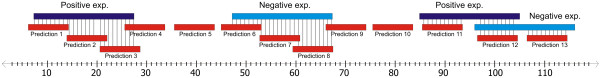
**Strategy employed to assess the predictors’ performance.** The scale bar represents the sequence of a theoretical protein of 120 amino acids. Dark blue rectangles represent one single epitope or a consensus of overlapping epitopes that were experimentally validated according to IEDB (Positive exp.); light blue rectangles represent one single non-immunogenic region or a consensus of overlapping non-immunogenic regions that were experimentally validated according to IEDB (Negative exp.); red rectangles represent the predicted epitopes from evaluated algorithms. For B cell prediction the predicted epitopes were considered true positive if they aligned with minimum coverage of 50% and 100% of identity with a Positive Exp. Region; for CD8+ T cell prediction the predicted epitopes were considered true positive if they aligned with minimum coverage of 87% and 100% of identity with a Positive Exp. region (Prediction 1, Prediction 2, Prediction 3 and Prediction 11). For B cell prediction the predicted epitopes were considered false positive if they aligned with minimum coverage of 50% and 100% of identity with a Negative Exp. Region; for CD8+ T cell prediction the predicted epitopes were considered false positive if they aligned with minimum coverage of 87% and 100% of identity with a Negative Exp. region (Prediction 6, Prediction 7, Prediction 8 and Prediction 13). Predictions were not considered during the analysis if they did not align with the parameters cited above (Prediction 4, Prediction 5, Prediction 9 and Prediction 10) or if they aligned with both Positive exp. and Negative exp. (Prediction 12).

When possible, in order to establish the ideal settings for protozoan epitope prediction, the algorithms parameters were scanned and evaluated in terms of AUC values. In this framework, the NetCTL score threshold parameter was ranged from 0.50 to 0.90. The NetCTL and NetMHC algorithms outputs were parsed and the data of 3,906 *in silico* predicted epitopes loaded into MySQL database. In order to evaluate the algorithm performances, predicted epitopes [see Additional file 1 and Additional file 2] were aligned against the consensus experimentally validated dataset for MHC class I epitopes. Figure 2 presents an overview of the benchmark approach undertaken in this study (see Methods Section for more details). In addition, we carried out a combined performance analysis using the best score threshold found for each methodology (Table 1).

**Table 1:** Algorithms performance evaluation for B cell epitope prediction and CD8+ T cells epitope prediction

**Algorithms**	**AUC values**
**CD8+ T cells epitope prediction**	
**NetCTL**	0.66
**NetMHC**	0.60
**NetCTL and NetMHC**	0.64
**B cell epitope prediction**	
**AAP12**	0.52
**BCPred12**	0.62
**BepiPred**	0.53
**AAP12 and BCPred12**	0.77
**AAP12 and BepiPred**	0.49
**BCPred12 and BepiPred**	0.58
**AAP12, BCPred12 and BepiPred**	0.57

**Figure 3:**
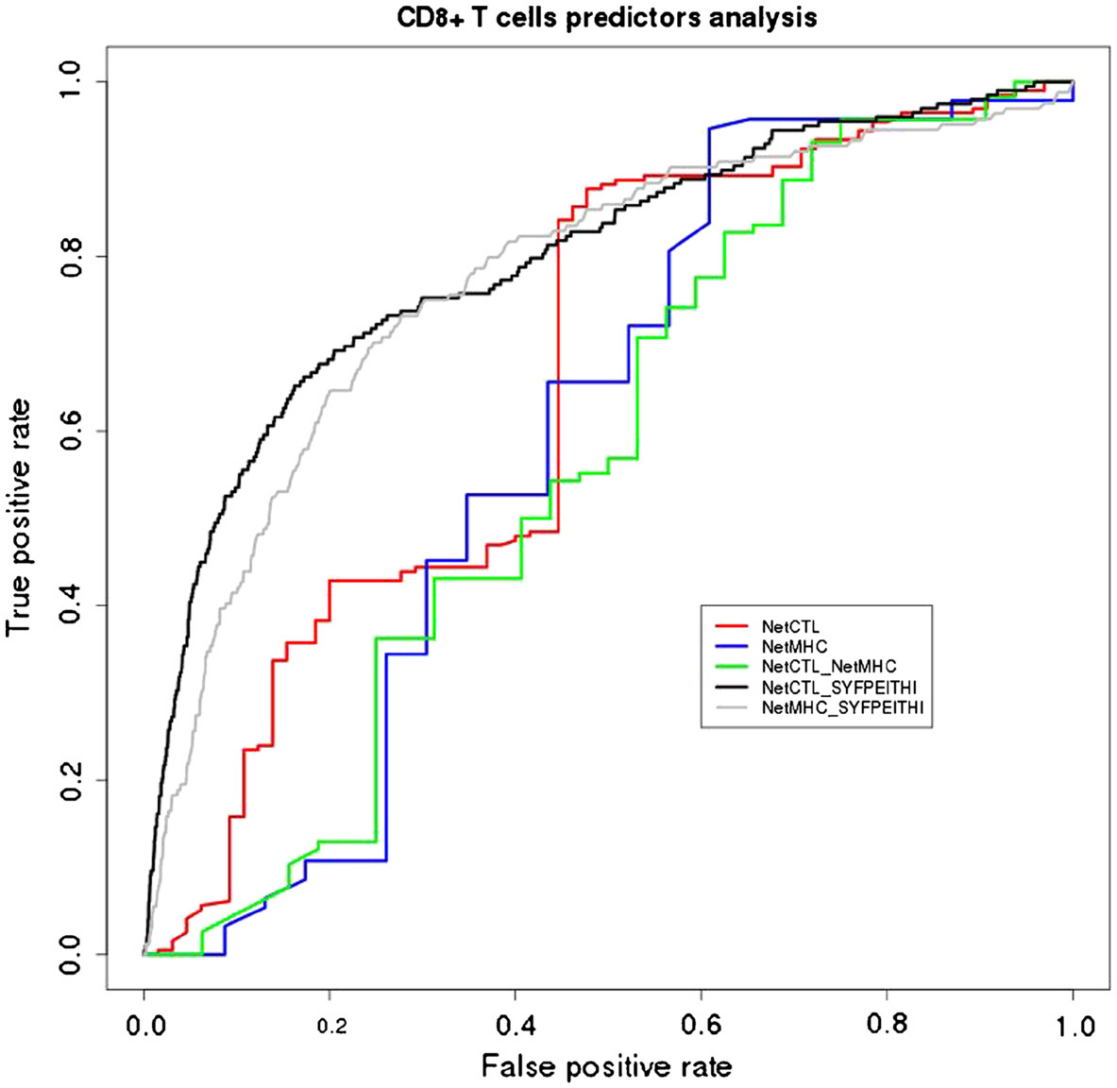
**ROC (Received Operating Characteristics) curve representing the performance of CD8+ T cells epitope predictors analyzed (NetCTL and NetMHC), from protozoan proteins database, and their combination.** In addition, the black and grey curves represent the performance of the same algorithms for human proteins database (SYFPEITHI) extracted from NetCTL homepage ( http://www.cbs.dtu.dk/suppl/immunology/CTL-1.2/syf.data.fsa).

The AUC performance measure obtained for NetCTL was 0.66 (for a score threshold of 0.50) and for NetMHC was 0.60 (score thresholds cannot be modified by the user). On the other hand, the combined performance of these algorithms produced an AUC value of 0.64 (Table 1, Figure 3).

### B-cell epitopes prediction

Following the same rational described above for MHC I algorithm selection, three currently available algorithms were chosen, BepiPred [24], BCPred12 [25] and AAP12 [26]. When possible, in order to establish the ideal settings for protozoan epitope prediction, the algorithms parameters were scanned and then evaluated in terms of AUC values. In this framework, the score thresholds parameter ranged from 0.15 to 0.90 for BepiPred and from 0.50 to 0.90 for AAP12 and BCPred12.

**Figure 4:**
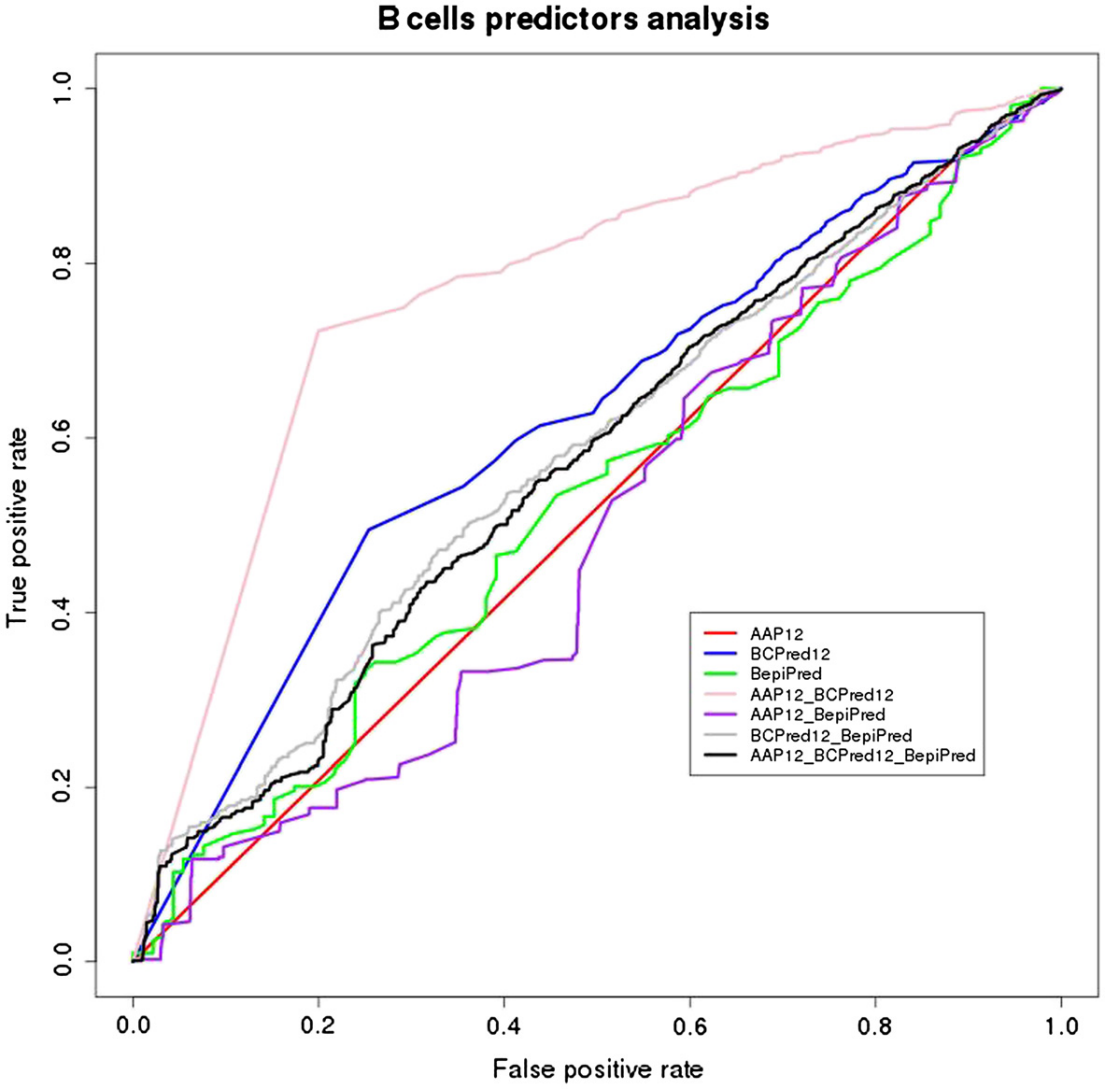
ROC (Received Operating Characteristics) curve representing the performance of B cells epitope predictors analyzed (AAP12, BCPred12 and BepiPred), from protozoan proteins database, and their combination.

Using the developed pipeline, the default algorithms outputs were parsed and the data of 187,187 *in silico* predicted epitopes [see Additional file 3, Additional file 4 and Additional file 5] loaded into the MySQL database. In order to evaluate the algorithm performances, predicted epitopes were aligned against the consensus experimentally validated dataset for B cell epitopes. Furthermore, we carried out a combined performance analysis using the best score threshold found for each methodology (Table 1, Figure 4).

The AUC performance measure obtained was: 0.53 for BepiPred using a threshold of 0.40; 0.52 for AAP12 using a threshold of 0.80; and 0.62 for BCPred12 using a threshold of 0.90 (Table 1). Regarding the combined performance analysis performed for these algorithms, the following results were found: 0.77 for AAP12 and BCPred12; 0.49 for AAP12 and BepiPred; 0.58 for BCPred12 and BepiPred; and 0.57 for AAP12, BCPred12 and BepiPred.

### Subcellular localization of proteins prediction

Regarding prediction of subcellular localization of proteins, three currently available algorithms were selected, WoLF PSORT [27], Sigcleave [28] and TargetP [11]. Using the developed pipeline, the default algorithms outputs were parsed and the data of 538 *in silico* predictions loaded into the MySQL database. In order to evaluate the algorithms performances, an experimental validated dataset of 180 proteins with described subcellular localization was loaded in addition to *in silico* predictions. Results show that WoLF PSORT was capable of correctly predicting 27/44 (61.36%) secreted proteins, Sigcleave, 30/44 (68.18%), and TargetP, 32/44 (72.73%), showing that the proportion of correctly predicted binders (sensitivity) was similar between the three algorithms (Table 2). Files containing predictions made by each algorithm are available as Additional files [see for WoLF PSORT, Additional file 6; for Sigcleave, Additional file 7; for TargetP, Additional file 8.

**Table 2:** Analyzed parameters for subcellular location of proteins

	**Subcellular location of proteins**		
	**WoLF PSORT**	**Sigcleave**	**TargetP**
**Sensitivity**	61.36% (27/44)	68.18% (30/44)	72.73% (32/44)
**Specificity**	93.38% (127/136)	76.47% (104/136)	79.41% (108/136)
**PPV**	74.29% (26/35)	48.39% (30/62)	53.33% (32/60)
**NPV**	88.11% (126/143)	88.14% (104/118)	90% (108/120)
**Accuracy**	85.39% (152/178)	74.44% (134/180)	77.78% (140/180)

**Figure 5:**
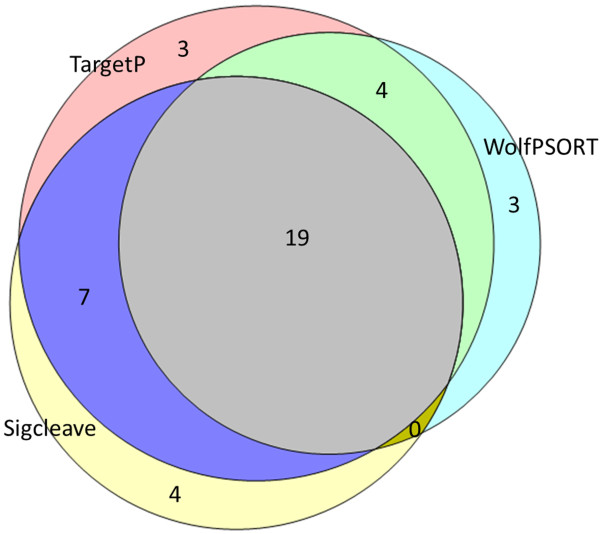
**Venn diagram showing the evaluation of the intersecting portion of predictions made by the tested algorithms for subcellular localization of proteins.** The evaluation considered just the proteins experimentally determined as extracellular (n=40).

The evaluation of the intersecting portion of predictions made by the tested algorithms showed that, from 40 protozoan proteins with extracellular localization experimentally determined, 19 (~48%) were correctly predicted by all three algorithms (Figure 5).

## Discussion

Despite of being a major public health problem in several countries, the life-threatening diseases caused by protozoan parasites represent a challenge in terms of vaccine development and nowadays there is no efficient vaccine against these parasites.

Epitope prediction by computational methods represents one of the most promising approaches to vaccine development, but there are several drawbacks in the process regarding trypanosomatid genomes. In this context, the lack of sufficiently large datasets of experimentally validated protozoan epitopes represents a serious limitation for validation of parasite *in silico* epitope prediction.

Several prediction methods were developed, but none of them had protozoan parasites data as training dataset (for some of them, protozoan parasites proteins represent only about 10% of the training dataset [24,25,29-32]) and, consequently, these results can be biased and should be treated with a grain of salt. The general wisdom is that the performance of epitope prediction methods critically depends on the dataset used for training and also on protein compositional bias. In addition, it is influenced by the evaluation criteria. Regarding epitope prediction in parasite genomes, these drawbacks are noteworthy considering that these organisms have a genome content that reflects proteins with a particular physicochemical profile and that are underrepresented in training datasets.

For this reason, we do not try to rank various prediction methods. Rather, we focus on the key concepts and ideas in the field. Thus, we evaluated algorithm performances focusing on parasites genomes. Comparison between algorithms was made in the basis of AUC (area under a ROC curve) values, which represent the probability that a randomly selected positive instance will score higher than a randomly selected negative instance [33].

Aiming at identifying a good set of tools for protozoan parasites epitope prediction and subcellular localization of proteins, we developed, in this work, a database approach in order to integrate and evaluate the combined performances of some open source currently available algorithms for MHC class I and B-cell epitope prediction, as well as for subcellular localization using protozoan parasites proteins and epitopes experimentally identified.

Concerning the epitope prediction, a database schema was developed and implemented integrating experimental validated data together with the information related to MHC I prediction (NetCTL and NetMHC algorithms) and B-cells prediction (BepiPred, AAP12 and BCPred12 algorithms).

The main source of experimental data was “Immune Epitope Database and Analysis Resource” (IEDB) ( http://www.immuneepitope.org/) [34], that currently represents the main source of linear and conformational epitopes data. Besides, IEDB uses a metric that takes into account the number of references, number of positive assays, and total number of assays for each epitope which is crucial to extract an experimentally validated epitope subset with a high level of confidence for the benchmark.

Regarding MHC I prediction, our AUC results indicate a little difference in the performances related with NetCTL and NetMHC algorithms, 0.66 and 0.60 respectively. If we consider that it is reported that the MHC class I prediction methods have achieved an accuracy that in many cases allows for AUC values in the range 0.95-0.99 [22], both algorithms didn′t achieve the expected performance. In fact, this is not the first time that underperformance of prediction algorithms is reported in literature. In a recent study, 167 9mer peptides from *Influenza A virus* were predicted as potential binders by NetMHC, and just 89 of them (53% of the pool) were confirmed as real binders [35]. Furthermore, the underrepresentation of protozoan proteins in the training datasets in general and the compositional bias certainly have a deep impact on epitope prediction methods and also in the benchmark. In fact, to highlight the different performances of tested algorithms in front of different datasets and exclude the influence of approach undertaken, we evaluated the algorithm performances under the same framework but with the human proteins dataset available for download from NetCTL website [36-38]. The results for both NetCTL and NetMHC algorithms were considerably better than the results obtained for protozoan dataset. The AUC value for NetCTL was 0.80 and for NetMHC was 0.77 (Figure 3). In addition, our performance evaluation does not include MHCII prediction since experimental data was insufficiently represented (data not shown). In practice, the prediction of MHC-peptide binding is far from perfect, but this fact does not preclude all the advances made in the last years in the field [21].

Regarding B-cell epitope prediction, our AUC results indicate a better performance for BCPred12 algorithms when compared to AAP12 and BepiPred (Table 1). Again the observed performances were inferior from those currently observed for B-cell epitope predictions [24]. This difference might be explained by same reasons which were just discussed for MHCI prediction. Also for B-cell epitope prediction, this is not the first report in literature of low epitope prediction performance [39].

Lafuente and Reche (2009) believe launching a Critical Assesment of Techniques for Epitope Prediction will benefit the field. Under this program, computational methods will be used for blind *de novo* prediction of peptides that are immunogenic from query proteins that, for evaluation purposes, has been experimentally screened [21]. Considering that and the results obtained by us, we do believe this approach will be useful to bring advances to epitope prediction area.

Despite of the shortcomings cited above, the combined performance analysis seems to be a promising approach. For B-cell algorithms, when the combined performance analysis was made, the best combination performance was found for AAP12 and BCPred12 that reached an AUC value of 0.77, which is within the expect range reported [24].

Seen in the light of the results obtained, the developed approach calls attention to several points: a) The general prediction models used by currently available algorithms cannot be used with the same performance for different protein subsets (especially true for protozoan parasites); b) The need for studies in which the algorithm performances are evaluated for underrepresented and compositional biased proteins subsets; and c) The combinatorial prediction approach can improve the epitope prediction performance.

Concerning the subcellular localization prediction, the database schema developed also integrated experimental and predicted data for subcellular localization of proteins. Experimental data was obtained from UniProt ( http://www.uniprot.org), and the *in silico* predictions made by WoLF PSORT, Sigcleave and TargetP algorithms. The result shows that there is not much difference, in terms of percentage of matches, between the tested algorithms. Nevertheless, the Venn diagram analysis related to true positives (extracellular localization) result shows that the tested algorithms match different proteins in the dataset, and the consensus prediction of the three algorithms would better define a protein located in the extracellular compartment.

## Conclusions

Considering the public health importance of the studied organisms and the lack of studies specifically addressing epitope and subcellular localization prediction in these parasites, our results suggest that the algorithm combinatorial approach employed in the developed database-driven epitope prediction methodology is capable of proposing the best set of tools for *in silico* epitope prediction in protozoan parasite genomes. Several drawbacks exist, but the present work will certainly speed up the process of data mining analysis and prediction of potential candidates for vaccine development.

## Methods

### Databases of experimentally tested epitopes

Two datasets of experimentally tested epitopes were built, one of B-cell epitopes and another of MHC-I epitopes. Parasite proteins experimental datasets where extracted from Immune Epitope Database and Analysis Resource (IEDB) [34,40,41]. The following criteria were adopted for epitope selection: a) from protozoan parasites; b) host organism must be mice or human. Selected epitopes were minimal epitopes, experimentally validated as immunogenic [see Additional file 9 and Additional file 10, for B and T-cell epitopes, respectively] or non-immunogenic [see Additional file 11 and Additional file 12, for B and T-cell non-immunogenic regions, respectively].

Furthermore, several overlapping epitopes anchored in the same protein region were observed. In order to have a non-redundant set of experimentally validated regions for each protein from dataset, make sense for us to use what we call “the consensus validated region”, which consists to cluster the overlapping epitopes in a unique consensus region called “experimentally validated consensus region”.

Thus, the dataset of B-cell experimental epitopes ended up with 312 proteins and 866 experimentally validated consensus regions including immunogenic and non-immunogenic [see Additional file 13 and Additional file 14, respectively]; for MHC-I epitopes, 81 proteins and 224 experimentally validated consensus regions including immunogenic and non-immunogenic assignments [see Additional file 15 and Additional file 16, respectively]. Furthermore, these data were used as input for the formatdb program (BLAST package) which prepares the sequences to be aligned as subject by the BLAST algorithm [42].

### Database of proteins with experimentally validated subcellular localization

Proteins with experimentally validated subcellular localization were obtained from UniProt. The search was done with the term “trypanosomatidae”, with the field “subcellular location” set to the confidence “experimental”, which retrieved 180 proteins with subcellular localization described experimentally [see Additional file 17 and Additional file 18]. This dataset was used to evaluate the three selected algorithms for subcellular localization prediction.

### Selection of prediction tools

The prediction algorithms were selected taking into account the possibility of being installed locally, and the reliability of their predictions reported on literature. The predictions of the following algorithms were evaluated: a) for MHCI epitope prediction: NetCTL [22,32,43] and NetMHC [23,31,44]; b) for B-cell epitope prediction: BepiPred [24] and BCPreds [25,26,29], which included two methodologies, AAP12 and BCPred12; c) for protein subcellular localization prediction, WoLF PSORT [27], Sigcleave [28] and TargetP [11].

### Score thresholds and allele used

Score thresholds used for CD8+ T cell epitope predictors ranged from 0.50 to 0.90 for NetCTL. We did not set the threshold value for NetMHC because this parameter is not variable in the NetMHC command line mode, so a unique value (0.426) was used for NetMHC. Concerning B cell epitope predictors, the score thresholds ranged from 0.50 to 0.90 for AAP12 and BCPred12 and from 0.15 to 0.90 for BepiPred.

For CD8+ T cell epitopes prediction, the human supertype A2 was the allele model used to scan MHC binding affinity. Other HLA alleles are present in IEDB, but regarding the protozoan proteins they are underrepresented in the non-redundant database used (see previous section). In addition, the supertype HLA-A2 is included in a group which is expressed in 88% of the population, what illustrates the relevance of the supertype used in this work.

### Development of parsers and algorithms

Parsers and algorithms were developed in PERL and SQL languages in order to extract the results obtained after running the programs and help to integrate all the results in the relational database.

### Construction of relational database

To aggregate all information generated during the development of the project we used MySQL as a Relational Database Management System (RDBMS) ( http://www.mysql.com). The use of a database system in this work represents a manner of getting a data receptacle or conceptual repository from which was possible to extract data correlation and results. The MySQL GUI Tools ( http://dev.mysql.com/downloads/gui-tools/5.0.html) were used as a graphical user interface for our MySQL database. The entity-relational model (ERM) was built using MySQL Workbench ( http://wb.mysql.com). For automatic data parsing and to load information into database, Perl scripts using DBI and BioPerl modules were developed. An overview of implemented workflow is presented in Figure 1.

### Analysis of the epitopes results

The developed methodology for result analyses was based on threshold dependent parameters and also on AUC performance analyses. After the identification of the regions of the source proteins which were assigned as consensus experimentally validated regions (immunogenic or non-immunogenic), and with the results of the predictions made by the tested algorithms stored on the constructed relational database, we identified the True Positives (TP) and False Positives (FP) hits. This information was employed in the AUC performance analysis.

In our methodology, we classified the predicted epitopes as TP or FP using the results produced from BLAST algorithm [42]. The following parameters were utilized to decide if a prediction was going to be considered as TP: 1) the local alignment between an epitope prediction (query) and an immunogenic consensus experimentally validated region (subject) had to have minimal query coverage (50% for B cell prediction and 87% for CD8+ T cell). In addition, the coverage cutoff was established based on a minimum size of B cell and CD8+ T cell found in the experimental epitopes database used (IEDB) which are 6 and 8 amino acids respectively. The NetCTL and NetMHC algorithms predict epitopes with 9 amino acids, thus 87% of coverage guarantees the minimum epitope alignment size of 8 amino acids. By the other hand, the AAP12 and BCPred12 algorithms predict epitopes with 12 amino acids, therefore 50% of coverage guarantees the minimum epitope alignment size of 6 amino acids. Regarding the BepiPred algorithm, since it predicts epitopes with variable sizes, only those with at least 6 amino acids were considered in the analysis. Specifically for predictions ranging from 6 to 11 amino acids, the coverage cutoff varied to guarantee a minimal amino acid alignment length of 6 residues. 2) The local alignment between an epitope prediction and a consensus experimentally validated region (immunogenic) had to have 100% of identity. This parameter was used to confirm that a given subject extracted from an alignment exactly matches with the real query. 3) Finally, in order to guarantee subject and query reciprocity the query name and the subject name must be the same. Using the same rational a prediction was considered FP, but the alignment analyses were made using as subject the non-immunogenic consensus experimentally validated region. Predicted epitopes that did not align with the parameters cited just above or if they aligned with both immunogenic and non-immunogenic consensus experimentally validated region were not considered for further analysis.

### Algorithms combined predictions

To perform the combined prediction we adopted the following rational: 1) for a given protein, the experimental regions are indexed and so, considering a protein (P) with three experimental validated regions, they would be named P1, P2 and P3; 2) if a given algorithm A predicts an epitope that matches with P2 for instance and another algorithm B predicts an epitope that also matches with P2 they are considered a combined prediction; 3) if a given algorithm A predicts an epitope that matches with P2 and another algorithm B predicts an epitope that matches with P1 or P3 they are not considered as a combined prediction.

Based on the above rules, the combined prediction score was calculated as the mean of the individual normalized scores of the original predictions. The score normalization was done as follows:

NS=(PS-MLS)/((MHS-MLS)/100), where:

NS = normalized score;

PS = prediction score;

MLS = methodology lowest score;

MHS = methodology highest score.

### Accuracy evaluation

A non-parametric performance measure was used to avoid the influence of arbitrary thresholds. In order to carry out an accuracy evaluation, we used the area under the ROC curve, or simply AUC, that aggregates the model’s behavior for all possible decision thresholds. The nonparametric estimate of the AUC [45] was calculated through an implemented GNU R package called ROCR [33].

### Analysis of the subcellular localization results

In order to analyze the subcellular localization prediction results, we determined the True Positives (TP), True Negatives (TN), False Positives (FP) and False Negatives (FN). This information was incorporated into a confusing error matrix that allowed the determination of parameters: sensitivity (Sn), specificity (Sp), positive predictive value (PPV), negative predictive value (NPV) and accuracy (Table 3).

**Table 3:** Parameters used in the analysis of the results

**Parameter**	**Brief description**	**Formula**
**Sensitivity (Sn)**	The proportion of correctly predicted binders	(a/(a + c))*100
**Specificity (Sp)**	The proportion of correctly predicted non-binders	(d/(b + d))*100
**Positive Predictive Value (PPV)**	The probability that a predicted binder will actually be a binder	(a/(a + b))*100
**Negative Predictive Value (NPV)**	The probability that a predicted non-binder will actually be a non-binder	(d/(c + d))*100
**Accuracy**	The proportion of correctly predicted peptides (both binders and non-binders)	((a + d)/(a+b+c+d)*100

a = True positive observations.

b = False positive observations.

c = False negative observations.

d = True negative observations.

## Competing interests

The authors declare that they have no competing interests.

## Authors’ contributions

DMR obtained the experimental tested epitopes and source proteins, selected the algorithms to be tested, run the selected algorithms, participated in the analysis of the results, and wrote the manuscript. AMR developed the algorithms for prediction performance analyses, participated in the analysis of the results and in the writing process of the manuscript. NJDO constructed and populated the relational database, developed algorithms in SQL language and participated in the analysis of the results; DMR, AMR and JCR developed parsers in PERL language that made possible results extraction and load the data into the relational database. ICAB monitored the analysis of the selected algorithms. ABR was involved with the conceptual design the study and RCO provided useful discussions for this work. JCR was involved in the drafting process of the manuscript and in the experimental and conceptual design of the study. All authors read and approved the final manuscript.

## Supplementary Material

Additional file 1 **Epitopes predicted by NetCTL.** This file in fasta format contains 2,657 epitopes predicted by NetCTL. Click here for file

Additional file 2 **Epitopes predicted by NetMHC.** This file in fasta format contains 1,249 epitopes predicted by NetCTL. Click here for file

Additional file 3 **Epitopes predicted by BepiPred.** This file in fasta format contains 5,450 epitopes predicted by BepiPred. Click here for file

Additional file 4 **Epitopes predicted by AAP12.** This file in fasta format contains 138,987 epitopes predicted by AAP12. Click here for file

Additional file 5 **Epitopes predicted by BCPred12.** This file in fasta format contains 42,750 epitopes predicted by BCPred12. Click here for file

Additional file 6 **Predictions made by WoLF PSORT.** This file contains predictions made by WoLF PSORT. Click here for file

Additional file 7 **Predictions made by Predictions made by Sigcleave.** This file contains predictions made by Sigcleave. Click here for file

Additional file 8 **Predictions made by TargetP.** This file contains predictions made by TargetP. Click here for file

Additional file 9 **B-cell minimal epitopes experimentally validated extracted from IEDB.** This file in fasta format contains 3,021 B-cell minimal epitopes from parasite proteins experimentally validated as immunogenic extracted from IEDB. Click here for file

Additional file 10 **CD8+ T cell minimal epitopes experimentally validated extracted from IEDB.** This file in fasta format contains 228 CD8+ T cell minimal epitopes from parasite proteins experimentally validated as immunogenic extracted from IEDB. Click here for file

Additional file 11 **B-cell non-immunogenic regions experimentally validated extracted from IEDB.** This file in fasta format contains 3,039 B-cell non-immunogenic regions from parasite proteins experimentally validated extracted from IEDB. Click here for file

Additional file 12 **CD8+ T cell non-immunogenic regions experimentally validated extracted from IEDB.** This file in fasta format contains 166 CD8+ T cell non-immunogenic regions from parasite proteins experimentally validated extracted from IEDB. Click here for file

Additional file 13 **B-cell immunogenic consensus regions experimentally validated.** This file in fasta format contains 607 B-cell immunogenic consensus regions from parasite proteins experimentally validated. Click here for file

Additional file 14 **B-cell non-immunogenic consensus regions experimentally validated.** This file in fasta format contains 243 B-cell non-immunogenic consensus regions from parasite proteins experimentally validated. Click here for file

Additional file 15 **CD8+ T cell immunogenic consensus regions experimentally validated.** This file in fasta format contains 140 CD8+ T cell immunogenic consensus regions from parasite proteins experimentally validated. Click here for file

Additional file 16 **CD8+ T cell non-immunogenic consensus regions experimentally validated.** This file in fasta format contains 84 CD8+ T cell cell non-immunogenic consensus regions from parasite proteins experimentally validated. Click here for file

Additional file 17 **Trypanosomatid proteins with experimentally validated subcellular localization extracted from Unitprot.** This file contains a list of 180 trypanosomatid proteins with its subcellular localization experimentally validated extracted from Uniprot. Click here for file

Additional file 18 **Trypanosomatid proteins in fasta format with experimentally validated subcellular localization.** This file in fasta format contains 180 trypanosomatid proteins with its subcellular localization experimentally validated. Click here for file
